# Comparison of Measured and Calculated Porosity Parameters of Woven Fabrics to Results Obtained with Image Analysis

**DOI:** 10.3390/ma17040783

**Published:** 2024-02-06

**Authors:** Živa Zupin, Veronika Štampfl, Tanja Nuša Kočevar, Helena Gabrijelčič Tomc

**Affiliations:** Department of Textiles, Graphic Arts and Design, Faculty of Natural Sciences and Engineering, University of Ljubljana, Snežniška ulica 5, 1000 Ljubljana, Slovenia; veronika.stampfl@ntf.uni-lj.si (V.Š.); tanja.kocevar@ntf.uni-lj.si (T.N.K.); helena.gabrijelcictomc@ntf.uni-lj.si (H.G.T.)

**Keywords:** woven fabrics, porosity, image analysis, statistical analysis

## Abstract

Porosity, the measure of the open spaces within a fabric structure, is a decisive factor in the performance of textiles. It influences breathability, permeability to liquids or gases, and suitability for various industries such as apparel, medical, and technical textiles. This study compares classical porosity calculation methods with non-destructive image analysis for 24 woven fabric samples that differ in density and weave pattern. Factors such as fabric density, weave pattern, illumination conditions, magnification, and the influence of the Otsu and Yen threshold algorithms were considered. The multifactor ANOVA statistical analysis shows that fabric density and weave pattern significantly influence porosity, with illumination playing an important role, while the threshold algorithm has a minor influence. A strong correlation is found between the actual fabric porosity and the results of the image analysis, except for double-sided illumination (reflective and transmissive), where the correlation is weakest. This comprehensive investigation provides valuable insights into the reliability of different porosity assessment approaches, which is essential for applications in various textile industries.

## 1. Introduction

Data on textile porosity are used in various fields, including materials science, medicine, and composite materials. In materials science, the porosity of textiles plays a crucial role in determining the permeability and thermal resistance of fabrics, influencing their suitability for specific applications [1,2]. In medicine, particularly in the development of vascular grafts and other types of implants, the porosity of textiles has been investigated as high porosity and structural stability are critical for favourable patient outcomes and effective incorporation into scar tissue [3,4]. Additionally, the porosity of textiles has implications in composite materials, as seen in the evaluation of fabric performance and antibacterial properties of 3D piezoelectric spacer fabrics, highlighting the significance of porosity in functional textile design [5]. The porosity and mechanical properties of textile-reinforced aerogel composites have been investigated, demonstrating the relevance of porosity in enhancing the thermal insulation and mechanical performance of advanced textile materials [6].

Several methods exist for assessing the porosity parameters of textile fabrics, such as: fluid flow methods, intrusion/extrusion methods, sieving methods, and geometric and optical methods. Despite the diversity of these methods, none of them provides universally satisfactory results across all application areas, as each comes with its own set of advantages and disadvantages [7].

In the field of textile analysis, image analysis techniques have been widely applied, offering a non-destructive and efficient method for obtaining various textile data [8]. These techniques have been used to determine geometric measurements of woven textiles, such as partial cover factors and yarn diameters, using automated tools and digital image analysis methods [9]. Furthermore, image analysis has also been employed for the investigation of textile deformations, with studies focusing on the metrological performance and uncertainty evaluation of these methods in assessing deformed textile materials [10]. The application of image analysis has extended to the determination of porosity, pore size, and pore size distribution, providing a novel approach to evaluating the characteristics of woven structures [11]. Research [12] has also found that methods using image analysis techniques to calculate textile porosity have easier reproducibility than experimental tests.

This shows the versatility and importance of image analysis in textile analysis, which covers various aspects from geometric measurements to the determination of deformation and porosity.

Many methods for calculating the three-dimensional porosity of textiles have been time-consuming, destructive, and prone to inaccuracies due to the deformation of the fabric during testing. Therefore, image analysis techniques have found their place in assessing the porosity of textiles instead [11].

The aim of our study was to compare the measured and calculated porosity parameters of specially designed and woven fabrics with the measured and calculated porosity values obtained by image analysis. The focus was to determine the correlation that can be used to predict fabric porosity parameters for a selected set of fabric construction parameters (yarn diameter, warp and weft density, and weave type) from the porosity results of image analysis by a computational comparison of porosity results (calculated and measured from real fabric and weave images) when using selected threshold algorithms, magnifications, and the type of sample illumination (during data acquisition). The novelty of the presented method and results lies in the number of analysed fabric samples and the carefully planned conditions for the acquisition and processing of the image information of the samples, such as illumination with reflection, transmission, and simultaneous reflection and transmission; magnification during the acquisition of the samples; and application algorithms, which have been empirically defined in several experiments as suitable for the determination of the porosity of selected fabric structures [13].

We analysed the influence of the following factors on the porosity of the fabrics: density, weave, illumination, magnification of the samples when photographed with a stereo microscope, and threshold algorithm. The statistical method of multifactor ANOVA was used to analyse the results. The results should confirm the usefulness of the image analysis method to obtain reliable information about the porosity of the woven fabric.

## 2. Materials and Methods

### 2.1. Materials

For the purpose of this study, 24 different woven fabric samples were produced. Cotton warp and weft yarns were of the same linear density for all fabrics, i.e., 17 × 2 tex, only warp being sized. Four different densities of warp and weft were chosen, i.e., warp densities 22 and 29.3 ends/cm, and weft densities 15 and 20 picks/cm, which resulted in four different densities of woven fabrics with 1 (22/15), 2 (22/20), 3 (29.3/15), and 4 (29.3/20) yarns/cm. Six different weave types were chosen: plain weave (PW), basket weave (BW), filling rib (R4/2), warp rib (R2/4), twill 1/3 (T1/3), and twill 2/2 (T2/2).

Physical characteristics of samples, measured warp and weft density, mass per square meter, and thickness were measured according to standards [14,15,16]. Set warp and weft density, measured warp and weft density, mass per square metre, and thickness of the fabrics are presented in Table 1 [17].

The diameter of the warp and weft threads was measured on captured images with all three types of illumination (transmitted, reflected, and both transmitted and reflected) and with both magnifications (5× (0.5) and 10× (1)). When measuring the yarn diameter, results were obtained with different illuminations and magnifications. Due to 240 measurements being taken for each yarn diameter, we could evaluate these averaged diameter values as reliable. Measured diameters are shown in Table 2.

### 2.2. Image Capture

Fabric images were acquired with a stereo microscope (Leica Microsystems GmbH, Wetzlar, Germany) under two magnifications, 5× (0.5) and 10× (1). Images were acquired using digiCamControl software, version 2.1.5.8. Three different illumination techniques were implemented and image data captured, i.e., under the sample (transmitted—T), above the sample (reflected—R), and simultaneously under and above the sample (transmitted and reflected—T + R). Five images were taken for each sample.

### 2.3. Classic Methods for Calculating the Porosity Parameters

The parameters often used for describing porosity are the number, diameter, volume, and distribution of pores. The parameters were calculated according to the equations presented in this section.

The total porosity—*ε*—is generally defined as a physical characteristic of woven fabrics which shows the portion of air volume in the total volume of a fabric [18,19]. It is expressed with Equation (1) in terms of corresponding densities:

(1) $$\varepsilon =\frac{V_{pores}}{V_{fabric}}\times 100=\left(1-\frac{\rho_{fabric}}{\rho_{fibre}}\right)\times 100=\left(1-\frac{M_{fabric}}{T_{fabric}\times 1000\times \rho_{fibre}}\right)\times 100,$$

where *ε* is the total porosity [%], *V_pores_* is the volume of pores in woven fabrics [cm^3^], and *V_fabric_* is the volume of woven fabric [cm^3^]. *ρ_fabric_* and *ρ_fibre_* represent physical densities of woven fabrics [g/cm^3^] and of fibres [g/cm^3^], respectively. *M_fabric_* is the fabric’s mass per square metre [g/m^2^], and *T_fabric_* is the fabric’s thickness [mm].

One of the parameters often used in dynamics of fluids when handling flow in non-circular tubes and channels is the so-called hydraulic diameter of pores *D_h_*. Different shapes of mainly rectangular pores in the woven fabrics can be translated into an equivalent diameter of an ideal round-shaped pore [17,20,21]. Hydraulic diameter is defined as a quotient between the pore cross-sectional area *A* and the wetted perimeter of the cross-section *P*, and can be calculated from Equations (2) and (3) for the most common shapes of macropores in woven fabrics. For squared cross-section of macropores, *D_h_* in µm is:

(2) $$D_{h}=\frac{4\times A_{pores}}{P_{pores}}=\frac{4a^{2}}{4a}=a,$$

where *A_pores_* is pores area, *P_pores_* is pores perimeter, and *a* is the length of the side of the macropores cross section in µm. For a rectangular cross-section of macropores, *D_h_* in µm is:

(3) $$D_{h}=\frac{4\times A_{pores}}{P_{pores}}=\frac{4ab}{2\left(a+b\right)}=\frac{2ab}{a+b},$$

where *a* and *b* are the lengths of the sides of the rectangular cross section of macropores in µm and can be calculated from known constructional parameters of woven fabrics—warp and weft density and warp and weft yarns diameter (*d_warp yarn_*/*d_weft yarn_*)—using the following equations:

(4) $$a=\frac{1}{warp\;density}-d_{warp\;yarn},$$

(5) $$b=\frac{1}{weft\;density}-d_{weft\;yarn}.$$

One of the important porosity properties is also the open area (*O_a_*) of woven fabrics, which is calculated from the cover factor (*C_f_*) of woven fabrics:

(6) $$C_{f}=\left(D_{1}d_{1}+D_{2}d_{2}-D_{1}d_{1}D_{2}d_{2}\right)\times 100,$$

where *C_f_* is the cover factor in %, *D*_1_ is the warp density in threads/cm, *D*_2_ is the weft density in threads/cm, *d*_1_ is the warp yarn diameter in cm, and *d_2_* is the weft yarn diameter in cm. Open area (*O_a_*) of woven fabric as a percentage is calculated as follows:

(7) $$O_{a}=100-C_{f}.$$

The equivalent average pore diameter was measured according to the methods developed by Dimitrovski [22]. The equivalent average pore diameter is defined as a diameter of cylindrical pore structures with a known number of pores that allow the same air permeability as the real woven fabric sample with the same number of pores and the same thickness.

The fluid flow through circular tubes in the range of laminar flow region with a low pressure drop results in the Hagen-Poiseuille equation shown in Equation (8):

(8) $$Q=\frac{\pi \cdot d^{4}\cdot n\cdot \Delta p}{128\cdot \mu \cdot l}=A\cdot \Delta p,$$

where *Q* is volume flow rate (cm^3^/s, cm^2^), *d* is diameter of pores (cm), *n* is the number of pores, Δ*p* is pressure drop (Pa), *μ* is the kinematic viscosity coefficient (Pas; in our case 1.8369 × 10^−5^), and *l* is the thickness of the fabric/length of the pores (cm).

Equation (8) shows that if the flow through a woven fabric is maintained in the laminar flow region with very low pressure, the pressure can identify the coefficient *A* and from its value, if the number of pores *n* is known, the equivalent average diameter of the pores can be calculated according to Equation (9):

(9) $$d_{e}=\sqrt[{4}]{\frac{128\cdot A\cdot \mu \cdot l}{\pi \cdot n}}$$

Measurements for the equivalent average pore diameter were made with an AIRTRONIC 4567 air permeability tester (Mesdan, Raffa, Italy) with upgraded software for instant calculation of the equivalent average diameter of the pores shown on the tester screen.

### 2.4. Image Analysis

The main objective of the image analysis was to detect pores within photographs of textiles and measure their properties. We wrote a Python script to perform image analysis, while NumPy [23] and OpenCV libraries [24] were used to perform basic actions within advanced functions. The script was designed to mimic the algorithms integrated into ImageJ software (version 1.53n), while it enabled automated processing of a larger image database. The database of 720 images was processed in 63.2 s, using an Apple MacBook Pro 2019 (2,6 GHz 6-Core Intel Core i7 processor, Intel UHD Graphics 630 1536 MB graphics card, 16 GB 2667 MHz DDR4 memory) and Visual Studio Code with Jupyter Notebooks.

The input images were thresholded using two different algorithms, Otsu [25] and Yen [26], where Otsu’s algorithm automatically sets the threshold based on the variance of the histogram to achieve the lowest intensity variance, while Yen’s algorithm uses quartiles to statistically determine the threshold. These two algorithms were selected based on their optimal performance in preliminary tests [13] among all the proposed algorithms of ImageJ software.

**Figure 1:**
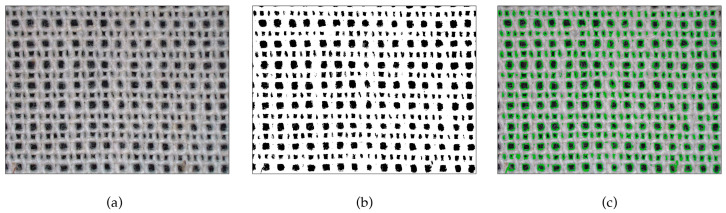
Steps of defining pores in the process of image analysis: (**a**) input image, (**b**) thresholded image, and (**c**) input image with drawn detected pores as overlay.

In each binary thresholded image, the pores were identified with an OpenCV function *findContours()*, while we eliminated the pores at the edges of the images, since they were not fully visible and would affect the final measurements. The set of found contours were then drawn on the initial images for visual assessment. These three steps are shown with an example in Figure 1.

During the image analysis, a scale was captured at both magnifications, which allowed us to determine that 1 cm corresponds to 432 px at magnification 0.5 and 702 px at magnification 1. Both values were squared in order to measure the surface areas in the desired units. Depending on the magnification used for each image, the corresponding coefficient was used.

The area of each pore was calculated with OpenCV *contourArea()* function and converted to squared millimetres using the coefficients. The initial results in squared pixels enabled fast calculation of the pore coverage as a percentage. To define the perimeter of each pore, the OpenCV *arcLength()* function was used and results again converted from pixels to millimetres. Calculated results for pores were gathered and averaged by the image.

### 2.5. Statistical Analysis and Results Evaluation

The experimental results were statistically analysed using a multifactorial ANOVA at 0.05 significance level. First, the results of the measured and calculated real fabrics were analysed, then we analysed the results of the image analyses of the woven fabrics.

First, we analysed results parameters measured and calculated on the real woven fabrics. Four independent variables—factors—were selected for the statistical analysis of the calculated and measured values of the porosity parameters of woven fabrics, as shown in Table 3. We wanted to find out which of the selected factors have significant influence on the calculated and measured values of the porosity parameters (open area, porosity, hydraulic diameter, and the equivalent average pore diameter). The magnification and light factors were selected because the yarn diameter was measured on the captured images at all three different illuminations and two magnifications, which may influence the calculated results.

Secondly, the experimental results of parameters measured and calculated using image analysis were statistically analysed by multifactor ANOVA. Five independent variables—factors—were selected for the statistical analysis of the image analysis of woven fabrics, as shown in Table 3. Each factor has two or three levels.

One of the aims of the research was to evaluate how the measured and calculated porosity parameters of the real woven fabrics correlate with the measured and calculated values of the image analysis. The statistical method of correlation was used to analyse the results. Correlation is a bivariate analysis that measures the strength of the relationship between two variables and the direction of the relationship.

To see how the porosity parameters of real woven fabrics and the results of the image analysis correlate with each other, the following comparison was made: open area, hydraulic diameter, equivalent pore diameter, and hydraulic diameter calculated from the results of the image analysis, and porosity of real fabrics and hydraulic diameter calculated from the results of the image analysis.

## 3. Results and Discussion

### 3.1. Multifactor ANOVA of Porosity Parameters Measured on Woven Fabrics

All results of the measured and calculated porosity parameters can be found in Appendix A.

The results of the statistical multifactor ANOVA analysis presented in Table 4 show that all four parameters tested are significant factors and influence the open area of woven fabrics. The statistically significant factors are fabric density, magnification, weave type and (least important) illumination.

It was expected that the density of the woven fabric would be the most important influencing factor. Woven fabrics with a higher warp and weft density have a lower open area, and vice versa. The results also show that the images of the fabrics for which a higher magnification was used also have a higher calculated open area, even though the fabrics are the same.

The open area (Table 4) is also influenced by the weave, but the weave has a smaller effect in the theoretical calculations as it is not actually taken into account. All other weaves except basket weave (BW) and twill 2/2 (T2/2) are statistically significant.

The statistical analysis of porosity (Table 5) confirms that only two factors, woven fabric density and weave, are statistically significant. This is understandable as the yarn diameter is not included in the calculation. The fabric density factor is statistically twice as significant as the weave factor.

The results show that the weaves with density 2 (22/20) and 3 (29.3/15) are not statistically significant and give almost the same results for the porosity of the fabrics. The statistical analysis also shows that there is no statistically significant difference between basket weave (BW) and twill 2/2 (T2/2).

The analysis of multifactor ANOVA confirms that all four factors have a statistically significant influence on the hydraulic diameter (*D_h_*) of the pores (Table 6). The order of the influencing factors is the same as for the open area. The most influential parameter is the density of the woven factors, followed by magnification, weave, and illumination. Fabric density is 30 times more important than the second factor, magnification. The result is understandable, as the hydraulic diameter is also calculated from the yarn diameter.

When analysing the weave type, the same trend can be seen: basket weave (BW) and twill 2/2 are not statistically significant. The reason for this is the construction of the weave and the interlacing of the threads. The highest hydraulic diameter value is found in the filling rib weave (R4/2), and the lowest hydraulic diameter value is found in the warp rib weave (R2/4).

When analysing the influence of four factors on the equivalent average pore diameter measured using the Dimitrovski method described in the experimental section, multivariate ANOVA shows that only woven fabric density and weave have a statistically significant influence on the equivalent average pore diameter, as is seen in Table 7. The weave factor is 13 times less important than density. If we observe the correlation between weave pattern and weave density, weave pattern has a very small influence on density, as shown in Table 1 (Set density and Measured warp and weft density). However, weave patterns mainly affect the positioning of the threads and the type of pores in the fabric, which influence the porosity of the fabric [27].

The smallest equivalent average pore diameter is found for fabrics in plain weave (PW), followed by twill 2/2 (T2/2), twill 1/3 (T1/3), warp rib (R2/4), and basket weave (BW), while the largest equivalent average pore diameter is found for filling rib (R4/2). This can be observed within the results found in Appendix A (Table A1).

### 3.2. Multifactor ANOVA of Porosity Parameters of Woven Fabrics Measured Using Image Analysis

All results of porosity parameters of woven fabrics measured and calculated using image analysis can be found in Appendix B.

**Figure 2:**
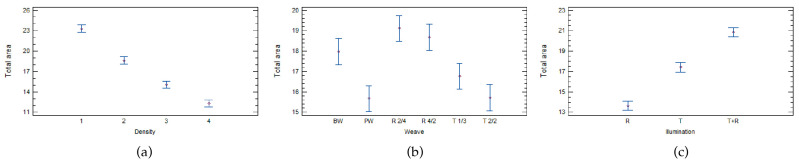
Influence of (**a**) density, (**b**) weave, and (**c**) illumination on the open area (total area) of woven fabrics measured using image analysis.

The statistical analysis of multifactor ANOVA in Table 8 shows that three of the five parameters tested are significant factors and that they actually influence the open area of woven fabrics. The most important factor is warp and weft density (D), followed by illumination, and the least important factor is weave (W). The warp and weft density (D) and illumination factors have almost the same influence on the open area of woven fabrics, while the weave factor is more than 10 times smaller, which is indicated by the F-ratio. Figure 2 shows the influence of each effect.

Figure 2a shows that woven fabrics with a lower density have a higher open area, which was expected. All samples’ densities are statistically significant in determining the open (total) area of the image samples.

The type of illumination also affects the open area of woven fabrics measured by image analysis. The highest open area is observed with a combination of transmissive and reflective (T + R) illumination. Woven fabrics illuminated with reflective illumination have the lowest open area (Figure 2c).

Statistically significant differences can be seen between some weaves in the woven fabrics (Figure 2b). Statistically, there is no significant difference between plain weave (PW) and twill 2/2 (T2/2), nor between warp rib (R2/4) and filling rib (R4/2). These weaves have a similar interweaving of threads and are therefore statistically similar.

**Figure 3:**
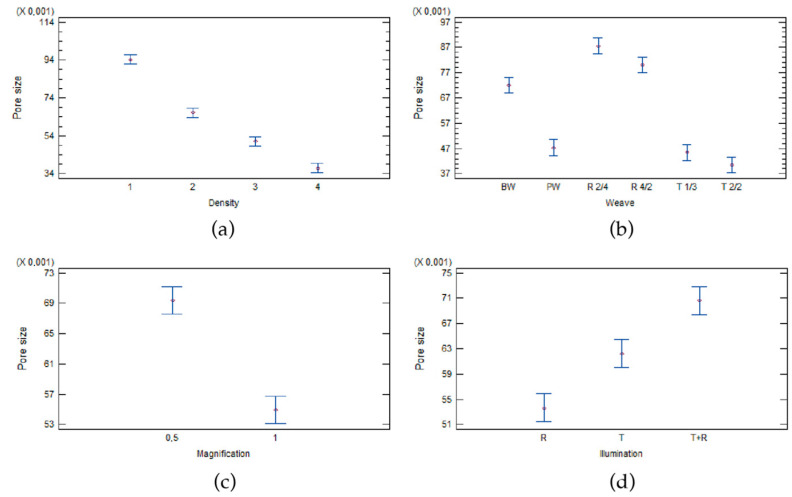
Influence of (**a**) density, (**b**) weave, (**c**) magnification, and (**d**) illumination on the pore size of woven fabrics measured using image analysis.

The pore size is also influenced by four of the five factors analysed in the experiment (Table 9). The most important factor is the density of the woven fabrics, followed by the weave of the woven fabrics and the magnification used for imaging, and the least influential factor on pore size is illumination. The factor threshold algorithm has no statistically significant influence on the pore size (Table 9), since P-Value is higher than 0.005. The results are shown in Figure 3.

Figure 3a shows that fabrics with lower fabric density have a higher pore size, as with calculated open area in woven fabrics.

Figure 3b shows that there are no significant differences between plain weave (PW), twill weave 1/3 (T1/3), and twill weave 2/2 (T2/2). These three weaves have approximately the same pore size, while there are statistical differences between basket weave (BW), warp rib (R2/4), and filling rib (R4/2).

The statistical analysis shows that magnification (Figure 3c) also has an influence on pore size. Woven fabrics at a lower magnification of 0.5 have a larger pore size, and fabrics at a higher magnification have a smaller pore size. Illumination also has an influence on pore size. There is a statistically significant difference between all three different types of illumination. The smallest pore size is observed with reflective illumination, and the largest with a combination of transmissive and reflective illumination.

The statistical analysis of multifactor ANOVA in Table 10 shows that all five parameters tested are statistically significant factors and influence the perimeter of pores of woven fabrics.

As expected, woven fabric density has the greatest influence on the perimeter of pores. As shown in the previous analysis, woven fabric density has the greatest influence on all porosity parameters, followed by these factors in order of importance: weave type, magnification, illumination, and threshold algorithm.

**Figure 4:**
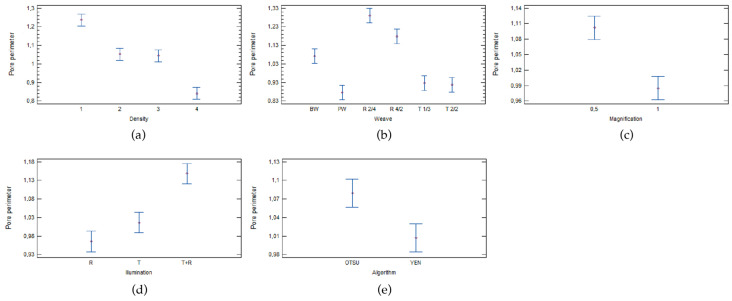
Influence of (**a**) density, (**b**) weave, (**c**) magnification, (**d**) illumination, and (**e**) threshold algorithm on the pore perimeter of woven fabrics measured using image analysis.

Figure 4a shows that woven fabrics with the lowest warp and weft density (density 1) have the highest perimeter of pores, and fabrics with the highest density (density 4) have the lowest, while there are no statistically significant differences between fabrics with a density of 2 and 3.

The statistical analysis also revealed that there are no statistically significant differences between weaves in plain weave (PW), twill weave 1/3 (T1/3), and twill weave 2/2 (T2/2). There are statistically significant differences between the other weaves, in order of significance: basket weave (BW), filling rib (R4/2), and warp rib (R2/4) (Figure 4b).

The values of the pore perimeter are also influenced by the magnification used for image analysis. The analyses show that woven fabrics which are captured with a magnification of 0.5 have a higher pore perimeter, while woven fabrics captured with a magnification of 1 have a slightly smaller pore perimeter (Figure 4c).

The statistical analysis also shows that illumination influences the average pore perimeter. There are no statistically significant differences between results for the samples acquired with reflective and transmissive illumination. Results for woven fabrics captured with all the illumination combinations have a statistically significant effect on average pore perimeter (Figure 4d).

The statistical effect on the average pore perimeter is also affected by the threshold algorithm. (Figure 4e) shows the statistical differences between the two threshold algorithms used. The values of the pore perimeter of the Otsu threshold algorithm are higher than the values of the Yen threshold algorithm.

When analysing the calculated hydraulic diameter from the results of the image analysis, the statistical analysis showed that the influential parameters are the same as when analysing the pore size and perimeter. All five factors are statistically important, as shown in Table 11.

The greatest statistical influence on the hydraulic diameter calculated from the image analysis is fabric density, followed by weave, magnification, illumination, and the least important factor, the threshold algorithm.

**Figure 5:**
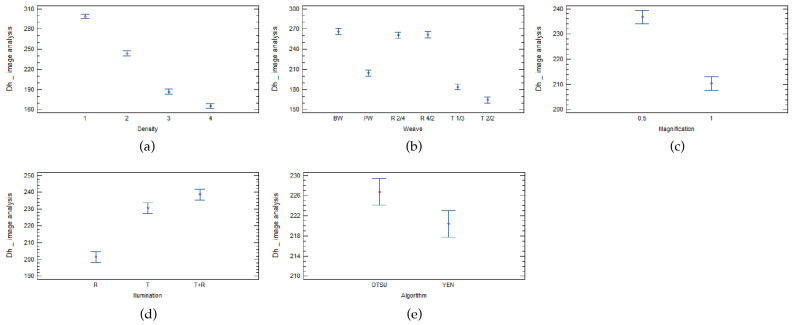
Influence of (**a**) density, (**b**) weave, (**c**) magnification, (**d**) illumination, and (**e**) threshold algorithm on the hydraulic diameter measured using image analysis.

The multifactor ANOVA analysis and Figure 5a show that all four woven fabric densities statistically significantly influence the fabric porosity. When analysing the weave, we obtained exactly the opposite result to that obtained when analysing the average perimeter of pores. The statistical analysis of the calculated hydraulic diameter of the pores from the image analysis shows that there is a statistically significant difference between plain weave (PW), twill 1/3 (T1/3), and twill 2/2 (T2/2), but no significant difference between basket weave (BW), filing rib, and warp rib (R2/4 and R4/2) (Figure 5b). From this we can conclude that the pores of woven fabrics are not the same size, although the theoretically calculated values of hydraulic diameter are the same.

The results when analysing the magnification factor are the same as when analysing pore size and pore perimeter. The results at lower magnification give larger pores and pore diameters (Figure 5c).

All three illumination types (reflection, transmission, and a combination of transmission and reflection) are statistically significant (Figure 5d).

Both threshold algorithms are statistically significant. Woven fabrics analysed with the Otsu threshold algorithm have a higher calculated hydraulic diameter (Figure 5e).

From the statistical analysis of multifactor ANOVA, it can be concluded that constructional parameters such as woven fabric density and weave are the most important factors influencing both the calculated porous parameters and those measured with image analysis. It was also found that the type of illumination, magnification, and threshold algorithm are important and influence the final image analysis measurement of the porous parameters of woven fabrics.

### 3.3. Results Correlation

To see how the measured and calculated porosity parameters of the real woven fabrics correlate with the measured and calculated values of the image analysis, we calculated the correlations between: the calculated open area measured on fabrics and the open area (total area) measured by image analysis; the calculated hydraulic diameter of the real fabrics and the hydraulic diameter calculated by image analysis; the measured equivalent pore diameter and the hydraulic diameter calculated by image analysis; and the correlation between the calculated porosity and the hydraulic diameter calculated by image analysis, which was the lowest correlation. The results are shown in Table 12.

Initially, all measurements were correlated, and it was found that the highest correlation exists between the measured average equivalent pore diameter and the hydraulic diameter calculated from image analysis measurements. The correlation coefficient between these two factors was 0.75. The correlation coefficient between the hydraulic diameter from real fabrics and the diameter calculated from image analysis was 0.68, and between the open areas 0.59. The lowest correlation coefficient was found between the porosity and the hydraulic diameter calculated from the results of image analysis.

The correlation between the parameters for individual groups of samples with the same illumination, magnification, and algorithm was performed. It was found that some samples in a group have a very high correlation with each other, such as the correlations between the calculated and the measured value of open area by image analysis. The highest correlation coefficient of 0.896 was achieved for samples with reflective illumination, magnification 1, and Otsu threshold algorithm (R 1 OTSU); and for samples with transmissive illumination, magnification 1, and Yen threshold algorithm (T 1 YEN).

A very low correlation coefficient, about 0.5, between the calculated open area and the open area value measured by image analysis is obtained with the combination of transmissive and reflective illumination and the Otsu threshold algorithm (T + R 0.5 OTSU and T + R 1 OTSU). Samples with the Yen threshold algorithm have a slightly higher correlation coefficient, about 0.7 (T + R 0.5 YEN and T + R 1 YEN).

The correlation coefficient between the hydraulic diameter calculated from the measured values on woven fabrics and the hydraulic diameter calculated from the values measured by image analysis averages 0.7 for all tested groups.

The highest correlation coefficient is achieved between the measured average equivalent pore diameter and the hydraulic diameter calculated from the values measured by image analysis. The highest correlation is found for samples with transmissive illumination and the Otsu threshold algorithm. The lowest correlation is obtained for samples with reflective illumination.

The lowest correlation coefficients are obtained between the porosity of the woven fabrics and the hydraulic diameter calculated from the values measured by image analysis. The correlation coefficient is around 0.5.

We also analyzed the correlation between individual weaves (Appendix C, Table A5). The results of the correlation between the analyzed porosity parameters show that weave type has a significant influence on the correlations between the results of the image analysis and the real fabric calculations. As expected, the highest levels of correlation are observed for plain weave, as this weave has only one type of pore and all pores in the fabric are approximately equal due to the interlacing of a warp and weft. For this reason, plain weave is normally used for theoretical models. The correlation coefficient was not less than 0.95 for all correlations we performed (open area (fabrics) and open (total) area (image), hydraulic diameter calculated from the fabrics and the image analysis, average pore diameter and hydraulic diameter calculated from the image analysis, and porosity of the fabrics and hydraulic diameter calculated from the image analysis).

For fabrics in which two or three threads float, the correlation between the analyzed porosity parameters measured and calculated on real fabrics and the parameters measured and calculated from the image analysis is lower. Other analyzed weaves have more than one type of pore present. The threads clump together so that the pores are not visible on the images. Such weaves are twill 1/3 and twill 2/2, as well as warp and filling rib. The threads are also close together in basket weave, but the correlations between the analyzed porosity parameters are sufficiently high. The correlation coefficient is 0.9 for the correlation of the open areas, and is lowest for the correlation of porosity and hydraulic diameter calculated from the values of the image analysis.

## 4. Conclusions

The results of the study show that the constructional parameters of woven fabrics—density and weave—are the most important factors influencing the porosity parameters we analysed (open area, porosity, hydraulic diameter, equivalent average pore diameter, and pore perimeter). The multifactor ANOVA shows that the largest influencing factor when analysing the porosity parameters of real woven fabrics is density. The porosity parameters (open area and hydraulic diameter) required for the calculation show that the illumination and magnification factors are also important, as the measured thread diameter influences the calculation.

The multifactor ANOVA also shows that constructional parameters (density and weave) influence the measured and calculated porosity parameters to a large extent. Illumination is also an important factor for image analysis. These three factors affect the open area of the captured fabric images; magnification factor also affects pore size, while all five influencing parameters affect pore perimeter and hydraulic diameter measured by image analysis.

From these results, it can be concluded that threshold algorithms that were selected for this research were not so significantly influential on the results as mechanical properties, illumination, and magnification when working with image analysis. This implies that automatic thresholding in Otsu and Yen thresholding algorithms are appropriate for analysis of textile samples. In addition, both algorithms proved to be the most optimal choice for determining the porosity of fabrics according to previous studies, where they allowed relevant data to be obtained and comparable porosity results on fabric images, which was not possible with the other tested algorithms [13].

The correlation method also shows some important features. The highest correlation is between the measured average equivalent pore diameter and the hydraulic diameter calculated from image analysis measurements. The lowest correlation coefficient was found between the porosity and the hydraulic diameter calculated from the results of the image analysis. From the correlation method, it can be concluded that the Yen threshold algorithm gives a slightly higher correlation than the Otsu algorithm. It was also found that illumination with a combination of transmissive and reflective illumination leads to lower correlation results.

As expected, when the individual weaves are compared to each other, the highest correlation coefficient is obtained between samples with plain weave, while the lowest correlation is obtained with twill weave.

Our research has confirmed that image analysis is a reliable and non-destructive method to analyse the porosity parameters of woven fabrics. The presented research shows the possibility of application to textile fabrics of various mechanical and structural parameters. The research results, with the presentation of correlations, offer the possibility of predicting the value of the porosity of fabrics without advanced calculations and time-consuming interventions in the fabric structures. The image analysis method is not only non-invasive but has also proven to be a statistically reliable method that allows a large amount of data to be calculated in a shorter time frame than acquiring traditional measurements.

## Figures and Tables

**Table 1:** Set and measured construction parameters for 24 woven fabric samples.

Weave Type	Sample	Set Density Warp/Weft	Measured Warp Density (ends/cm)	Measured Weft Density (picks/cm)	Mass (g/m^2^)	Thickness (mm)
Plain weave (PW)	1	22/15	21	15	143.91	0.439
	2	22/20	21	20	166.59	0.438
	3	29.3/15	28	15	180.41	0.468
	4	29.3/20	28.5	20	209.50	0.514
Basket weave (BW)	5	22/15	21	15	140.52	0.506
	6	22/20	22	20	160.90	0.531
	7	29.3/15	29	15	173.12	0.604
	8	29.3/20	29	20	195.98	0.567
Filling rib 4/2 (R4/2)	9	22/15	20	15	138.14	0.591
	10	22/20	21	20	160.60	0.558
	11	29.3/15	28	15	171.53	0.620
	12	29.3/20	28	20	196.30	0.605
Warp rib 2/4 (R2/4)	13	22/15	22	14	143.77	0.549
	14	22/20	22	19.5	163.89	0.557
	15	29.3/15	30	15	179.93	0.471
	16	29.3/20	31	20	202.52	0.480
Twill 1/3 (T1/3)	17	22/15	22	15	141.36	0.565
	18	22/20	21	20	162.24	0.558
	19	29.3/15	29.5	15	176.61	0.596
	20	29.3/20	29.5	20.5	198.11	0.586
Twill 2/2 (T2/2)	21	22/15	21	15	141.78	0.508
	22	22/20	21.5	20	160.66	0.504
	23	29.3/15	29	15	173.34	0.604
	24	29.3/20	29	20	197.48	0.568

**Table 2:** Diameter of warp and weft under different illuminations (transmitted (T), reflected (R), and both transmitted and reflected (T + R)) and magnifications.

Illumination	Yarn Diameter (mm)							
	Warp				Weft			
	0.5		1		0.5		1	
	Avg	CV	Avg	CV	Avg	CV	Avg	CV
T	0.297	14.90	0.285	13.78	0.297	15.65	0.285	13.94
R	0.301	13.17	0.289	13.87	0.301	15.21	0.289	13.10
T + R	0.298	17.30	0.289	15.92	0.298	14.22	0.289	15.03

**Table 3:** Experimental design diagram.

Influencing Parameter	Factor	Level
Weave	W	Plain weave (PW)
		Basket weave (BW)
		Filling rib 4/2 (R4/2)
		Warp rib 2/4 (R2/4)
		Twill 1/3 (T1/3)
		Twill 2/2 (T2/2)
Warp/weft density	D	22/15 (1)
		22/20 (2)
		29/15 (3)
		29/20 (4)
Illumination	L	Transmissive (T)
		Reflective (R)
		Transmissive and reflective (T + R)
Threshold algorithms	Alg	Otsu (OTSU)
		Yen (YEN)

**Table 4:** Effect of factors on open area of woven fabrics.

Effects	Sum of Squares	Degrees of Freedom	Mean Squares	F-Ratio	*p*-Value
Illumination	26.59	2	13.30	36.74	0.0000
Magnification	116.70	1	116.70	322.44	0.0000
Weave	92.70	5	18.54	51.22	0.0000
Density	5155.10	3	1718.37	4747.89	0.0000
Residual effects	47.77	132	0.36	/	/
Total (corrected)	5438.86	143	/	/	/

**Table 5:** Effect of factors on porosity of woven fabrics.

Effects	Sum of Squares	Degrees of Freedom	Mean Squares	F-Ratio	*p*-Value
Illumination	0.0	2	0.0	0.00	1.0000
Magnification	0.0	1	0.0	0.00	1.0000
Weave	627.0	5	125.4	78.9	0.0000
Density	669.3	3	223.1	140.4	0.0000
Residual effects	209.8	132	1.6	/	/
Total (corrected)	1506.1	143	/	/	/

**Table 6:** Effect of factors on hydraulic diameter of pores (*D_h_*) of woven fabrics.

Effects	Sum of Squares	Degrees of Freedom	Mean Squares	F-Ratio	*p*-Value
Illumination	1006.7	2	503.4	10.1	0.0001
Magnification	6920.8	1	6920.8	138.3	0.0000
Weave	14,395.9	5	2879.2	57.5	0.0000
Density	659,770.0	3	219,923.0	4394.4	0.0000
Residual effects	6606.1	132	50.105	/	/
Total (corrected)	688,700.0	143	/	/	/

**Table 7:** Effect of factors on equivalent average pore diameter of woven fabrics.

Effects	Sum of Squares	Degrees of Freedom	Mean Squares	F-Ratio	*p*-Value
Illumination	0.0	2	0.0	0.00	1.0000
Magnification	0.0	1	0.0	0.00	1.0000
Weave	27,659.5	5	5531.9	87.8	0.0000
Density	223,669.0	3	74,556.3	1183.218	0.0000
Residual effects	8317.8	132	63.0	/	/
Total (corrected)	259,646.0	143	/	/	/

**Table 8:** Effect of influencing factors on open area measured using image analysis.

Effects	Sum of Squares	Degrees of Freedom	Mean Squares	F-Ratio	*p*-Value
Algorithm	1.18	1	1.18768	0.12	0.7326
Illumination	2497.34	2	1248.67	124.14	0.0000
Magnification	2.40	1	2.40	0.24	0.6256
Weave	530.28	5	106.06	10.54	0.0000
Density	4906.89	3	1635.63	162.61	0.0000
Residual	2766.15	275	10.06	/	/

**Table 9:** Effect of influencing factors on pore size measured using image analysis.

Effects	Sum of Squares	Degrees of Freedom	Mean Squares	F-Ratio	*p*-Value
Algorithm	0.001	1	0.001	3.70	0.0554
Illumination	0.014	2	0.007	28.82	0.0000
Magnification	0.015	1	0.015	61.86	0.0000
Weave	0.098	5	0.020	81.32	0.0000
Density	0.130	3	0.043	179.69	0.0000
Residual	0.066	275	0.0002	/	/

**Table 10:** Effect of influencing factors on perimeter of pores measured using image analysis.

Effects	Sum of Squares	Degrees of Freedom	Mean Squares	F-Ratio	*p*-Value
Algorithm	0.38	1	0.38	9.95	0.0018
Illumination	1.71	2	0.85	22.50	0.0000
Magnification	0.99	1	0.99	26.15	0.0000
Weave	6.54	5	1.31	34.48	0.0000
Density	5.64	3	1.88	49.55	0.0000
Residual	10.44	275	0.04	/	/

**Table 11:** Effect of influencing factors on hydraulic diameter measured using image analysis.

Effects	Sum of Squares	Degrees of Freedom	Mean Squares	F-Ratio	*p*-Value
Algorithm	2897.6	1	2897.6	5.63	0.0183
Illumination	73,228.8	2	36,614.4	71.20	0.0000
Magnification	49,765.8	1	49,765.8	96.78	0.0000
Weave	483,866.0	5	96,773.1	188.19	0.0000
Density	775,445.0	3	258,482.0	502.65	0.0000
Residual	140,900.0	274	514.2	/	/

**Table 12:** Correlation coefficients between parameters measured on real fabrics and captured images.

Parameter Correlation	Transmission				Reflection				Transmission—Reflection			
	Otsu		Yen		Otsu		Yen		Otsu		Yen	
	0.5	1	0.5	1	0.5	1	0.5	1	0.5	1	0.5	1
Open area (fabrics)— Open area (image)	0.746	0.805	0.889	0.896	0.853	0.896	0.860	0.862	0.494	0.564	0.687	0.690
*D_h_* (fabrics)—*D_h_* (image)	0.744	0.764	0.747	0.753	0.728	0.731	0.730	0.743	0.737	0.736	0.734	0.698
Equivalent pore diameter (fabrics)—*D_h_* (image)	0.816	0.813	0.802	0.791	0.744	0.771	0.775	0.804	0.783	0.833	0.772	0.784
Porosity (fabrics)—*D_h_* (image)	0.606	0.555	0.512	0.463	0.494	0.502	0.502	0.518	0.563	0.594	0.458	0.441

## Data Availability

Data are contained within the article.

## Appendix A

**Table A1:** Calculation of porosity parameters for woven fabrics: porosity, equivalent pore diameter, open area, and hydraulic diameter calculated with yarn diameter measured at different illuminations.

				Transmission				Reflection				Transmission + Reflection			
				0.5		1		0.5		1		0.5		1	
Weave	Density	Porosity [%]	Diameter [μm]	Open Area [%]	*D_h_* [μm]	Open Area [%]	*D_h_* [μm]	Open Area [%]	*D_h_* [μm]	Open Area [%]	*D_h_* [μm]	Open Area [%]	*D_h_* [μm]	Open Area [%]	*D_h_* [μm]
PW	20/15	78.45	223.90	21.04	242.18	22.87	254.41	20.12	236.72	22.15	249.94	19.90	237.13	21.68	247.95
	20/22	74.98	180.80	15.50	191.64	17.10	201.50	14.55	185.82	16.41	197.39	14.07	182.99	15.83	194.00
	29.3/15	74.64	169.90	9.45	103.96	11.52	121.48	8.61	97.47	10.77	115.62	8.80	101.20	10.49	114.55
	29.3/20	73.21	126.10	6.35	85.72	8.01	100.80	5.63	79.81	7.38	95.67	5.66	82.40	7.08	94.33
BW	20/15	81.72	256.60	21.04	242.18	22.87	254.41	20.12	236.72	22.15	249.94	19.90	237.13	21.68	247.95
	20/22	80.08	204.70	14.28	178.57	15.89	188.82	13.37	172.91	15.21	184.68	12.95	170.84	14.66	181.65
	29.3/15	81.13	205.50	7.79	85.20	9.90	103.61	6.97	78.44	9.14	97.49	7.21	82.54	8.89	96.48
	29.3/20	77.28	164.50	5.74	77.96	7.40	93.60	5.04	71.91	6.78	88.32	5.10	74.83	6.49	87.06
R 4/2	20/15	84.62	282.80	22.70	262.96	24.49	274.61	21.76	257.51	23.77	270.26	21.49	257.35	23.28	268.05
	20/22	81.05	215.80	15.50	191.64	17.10	201.50	14.55	185.82	16.41	197.39	14.07	182.99	15.83	194.00
	29.3/15	81.80	215.10	9.45	103.96	11.52	121.48	8.61	97.47	10.77	115.62	8.80	101.20	10.49	114.55
	29.3/20	78.66	168.40	6.96	93.39	8.62	107.94	6.23	87.60	7.98	102.94	6.22	89.85	7.66	101.51
R 2/4	20/15	82.76	277.40	20.41	229.41	22.32	242.90	19.50	223.84	21.58	238.13	19.39	225.28	21.17	236.51
	20/22	80.64	206.00	14.79	183.24	16.42	193.70	13.88	177.65	15.74	189.55	13.49	175.93	15.21	186.67
	29.3/15	74.84	175.10	6.13	66.66	8.28	86.01	5.32	59.60	7.52	79.61	5.62	64.05	7.30	78.63
	29.3/20	72.24	143.10	3.30	45.93	4.98	64.12	2.66	39.11	4.37	58.14	2.85	43.28	4.16	57.10
T 1/3	20/15	83.53	265.80	19.39	221.67	21.25	234.53	18.48	216.16	20.52	229.92	18.32	217.13	20.09	228.13
	20/22	80.87	203.30	15.50	191.64	17.10	201.50	14.55	185.82	16.41	197.39	14.07	182.99	15.83	194.00
	29.3/15	80.50	181.90	6.96	75.90	9.09	94.78	6.14	68.99	8.33	88.52	6.42	73.27	8.10	87.52
	29.3/20	77.74	139.80	4.94	69.36	6.57	85.28	4.27	63.22	5.96	79.93	4.35	66.31	5.69	78.70
T 2/2	20/15	81.62	242.60	21.04	242.18	22.87	254.41	20.12	236.72	22.15	249.94	19.90	237.13	21.68	247.95
	20/22	79.01	194.40	14.89	185.14	16.49	195.18	13.96	179.41	15.81	191.07	13.51	176.96	15.25	187.86
	29.3/15	81.12	188.30	7.79	85.20	9.90	103.61	6.97	78.44	9.14	97.49	7.21	82.54	8.89	96.48
	29.3/20	77.11	145.40	5.74	77.96	7.40	93.60	5.04	71.91	6.78	88.32	5.10	74.83	6.49	92.49

## Appendix B

**Table A2:** Measurement and calculation of porosity parameters for woven fabrics using image analysis and reflectance illumination: open area, average pore size, and perimeter and hydraulic diameter at different threshold algorithms and magnifications.

		Otsu								Yen							
		0.5				1				0.5				1			
Weave	Density	Open Area [%]	Size [mm^2^]	Perimeter [mm]	*D_h_* [μm]	Open Area [%]	Size [mm^2^]	Perimeter [mm]	*D_h_* [μm]	Open Area [%]	Size [mm^2^]	Perimeter [mm]	*D_h_* [μm]	Open Area [%]	Size [mm^2^]	Perimeter [mm]	*D_h_* [μm]
PW	20/15	18.06	0.08	1.15	263.99	18.45	0.06	1.13	220.89	19.73	0.08	1.19	275.80	21.17	0.07	1.18	241.68
	20/22	13.81	0.04	0.85	207.13	13.65	0.04	0.81	184.27	12.92	0.04	0.82	200.82	13.14	0.04	0.80	180.93
	29.3/15	11.33	0.04	0.85	193.69	11.44	0.03	0.77	164.19	9.31	0.04	0.82	180.72	9.57	0.03	0.73	151.64
	29.3/20	10.04	0.03	0.69	157.27	8.06	0.02	0.57	117.72	3.97	0.02	0.55	126.61	3.70	0.01	0.48	91.72
BW	20/15	19.04	0.15	1.55	391.65	18.35	0.08	0.94	320.85	22.67	0.12	1.23	377.06	22.81	0.06	0.82	304.79
	20/22	15.60	0.12	1.41	346.51	15.38	0.08	1.08	299.35	18.53	0.11	1.21	351.55	19.18	0.06	0.83	291.82
	29.3/15	11.85	0.06	1.15	197.38	11.24	0.03	0.85	164.33	12.44	0.06	1.16	198.78	12.38	0.04	0.88	167.27
	29.3/20	9.45	0.05	0.97	212.86	9.22	0.03	0.74	173.58	9.66	0.05	0.96	213.64	9.57	0.03	0.73	174.50
R 4/2	20/15	21.32	0.14	1.61	325.54	20.38	0.11	1.53	283.72	25.76	0.16	1.72	354.81	25.55	0.13	1.53	314.14
	20/22	16.96	0.08	1.27	292.24	17.13	0.07	1.22	236.83	18.28	0.09	1.32	313.20	19.30	0.08	1.34	262.30
	29.3/15	11.61	0.05	0.98	197.04	10.75	0.04	0.90	185.33	11.91	0.05	0.99	191.75	11.27	0.04	0.92	187.79
	29.3/20	9.45	0.03	0.77	191.99	9.99	0.03	0.80	159.74	7.53	0.03	0.69	180.23	8.87	0.03	0.74	148.72
R 2/4	20/15	20.25	0.15	1.78	339.06	20.95	0.10	1.43	298.15	24.81	0.15	1.72	369.14	27.08	0.10	1.26	328.65
	20/22	16.01	0.10	1.32	255.37	15.84	0.07	1.17	218.66	18.90	0.11	1.35	266.94	19.44	0.08	1.22	235.69
	29.3/15	12.47	0.07	1.35	203.24	13.53	0.05	1.17	170.94	11.73	0.06	1.35	205.35	13.86	0.05	1.17	174.59
	29.3/20	9.96	0.05	1.01	168.15	9.89	0.03	0.85	153.33	8.48	0.04	0.99	151.79	8.37	0.03	0.80	144.48
T 1/3	20/15	18.14	0.06	1.06	239.57	18.76	0.05	0.92	206.38	19.76	0.07	1.08	245.97	22.68	0.06	1.00	223.45
	20/22	13.92	0.04	0.88	184.80	13.25	0.03	0.75	153.73	10.59	0.03	0.84	165.57	10.61	0.02	0.69	140.84
	29.3/15	11.24	0.03	0.80	155.07	10.60	0.02	0.65	128.93	6.21	0.02	0.73	133.95	6.69	0.02	0.58	110.15
	29.3/20	15.93	0.04	1.00	146.65	12.33	0.02	0.67	108.88	2.51	0.01	0.45	88.71	2.85	0.01	0.39	75.74
T 2/2	20/15	17.35	0.06	1.05	215.85	17.24	0.04	0.92	182.08	16.21	0.05	1.01	209.58	17.46	0.04	0.94	183.79
	20/22	14.19	0.03	0.81	171.76	14.00	0.03	0.77	142.64	9.44	0.02	0.69	144.44	10.04	0.02	0.63	120.05
	29.3/15	12.38	0.03	0.90	136.40	12.07	0.02	0.77	112.79	4.48	0.02	0.63	98.77	4.91	0.01	0.50	79.24
	29.3/20	14.79	0.03	0.94	138.13	13.33	0.02	0.73	106.45	2.26	0.01	0.41	77.53	2.13	0.00	0.34	58.83

**Table A3:** Measurement and calculation of porosity parameters for woven fabrics using image analysis and transmissive illumination: open area, average pore size, perimeter, and hydraulic diameter at different threshold algorithms and magnifications.

		Otsu								Yen							
		0.5				1				0.5				1			
Weave	Density	Open Area [%]	Size [mm^2^]	Perimeter [mm]	*D_h_* [μm]	Open Area [%]	Size [mm^2^]	Perimeter [mm]	*D_h_* [μm]	Open Area [%]	Size [mm^2^]	Perimeter [mm]	*D_h_* [μm]	Open Area [%]	Size [mm^2^]	Perimeter [mm]	*D_h_* [μm]
PW	20/15	20.60	0.08	1.19	278.86	22.19	0.08	1.18	266.40	23.58	0.09	1.20	295.91	26.60	0.09	1.17	292.40
	20/22	16.53	0.05	0.90	218.37	17.09	0.05	0.89	209.59	17.80	0.05	0.92	225.39	18.63	0.05	0.91	217.82
	29.3/15	13.76	0.04	0.85	205.11	13.85	0.04	0.82	186.82	13.27	0.04	0.85	202.87	13.76	0.04	0.81	186.36
	29.3/20	10.09	0.03	0.65	158.16	10.80	0.02	0.63	142.51	6.95	0.02	0.61	143.96	7.76	0.02	0.58	128.78
BW	20/15	21.83	0.12	1.23	377.00	19.57	0.07	0.88	318.31	25.99	0.10	1.19	350.74	24.09	0.07	0.94	308.68
	20/22	17.38	0.09	1.10	336.88	17.58	0.06	0.83	294.61	20.52	0.08	0.98	317.16	22.17	0.06	0.81	276.97
	29.3/15	15.79	0.06	1.26	206.16	15.15	0.05	0.98	183.67	16.12	0.07	1.27	207.11	16.81	0.05	1.11	191.35
	29.3/20	11.72	0.05	0.89	213.07	11.79	0.04	0.77	211.09	11.67	0.05	0.89	212.99	12.40	0.04	0.77	211.33
R 4/2	20/15	24.63	0.15	1.60	367.47	24.08	0.14	1.55	326.63	30.30	0.16	1.54	377.27	30.44	0.12	1.24	335.53
	20/22	19.93	0.09	1.30	323.37	19.82	0.09	1.36	294.23	22.82	0.11	1.40	339.19	22.88	0.10	1.45	308.02
	29.3/15	13.45	0.06	1.03	252.69	13.65	0.05	1.00	210.54	15.46	0.06	1.03	240.65	15.64	0.05	0.96	205.23
	29.3/20	11.79	0.04	0.81	245.07	11.83	0.04	0.88	196.50	12.67	0.04	0.84	238.65	12.58	0.04	0.90	191.27
R 2/4	20/15	23.04	0.15	1.64	385.97	22.73	0.11	1.30	353.60	27.48	0.14	1.47	410.71	28.66	0.10	1.20	374.65
	20/22	19.36	0.10	1.29	286.38	19.49	0.09	1.19	259.46	22.91	0.11	1.27	307.55	23.83	0.09	1.11	283.40
	29.3/15	18.34	0.09	1.46	222.98	17.00	0.06	1.20	213.27	15.59	0.08	1.41	233.17	15.75	0.06	1.17	223.13
	29.3/20	14.25	0.06	1.02	196.92	13.96	0.04	0.90	187.15	12.80	0.06	1.01	204.14	12.60	0.04	0.91	193.24
T 1/3	20/15	23.28	0.08	1.13	271.60	23.02	0.06	1.05	243.35	24.92	0.08	1.17	278.31	26.38	0.07	1.15	259.66
	20/22	17.85	0.05	0.92	205.29	18.85	0.04	0.90	191.23	15.17	0.04	0.85	195.89	17.12	0.04	0.86	183.15
	29.3/15	19.55	0.05	1.06	196.28	17.98	0.04	0.87	168.36	9.55	0.03	0.76	153.05	9.40	0.02	0.63	132.04
	29.3/20	19.59	0.06	1.16	193.15	18.61	0.04	1.09	163.92	5.76	0.02	0.52	124.65	5.03	0.01	0.46	95.66
T 2/2	20/15	22.98	0.08	1.19	253.45	22.93	0.06	1.11	226.52	23.14	0.08	1.20	254.33	23.10	0.06	1.11	227.58
	20/22	18.74	0.05	0.93	202.13	18.48	0.04	0.90	179.42	15.04	0.04	0.81	184.54	15.01	0.03	0.79	160.97
	29.3/15	18.71	0.05	1.16	171.13	18.93	0.04	1.05	160.77	7.35	0.02	0.71	113.14	7.97	0.02	0.62	103.45
	29.3/20	16.76	0.04	1.02	155.46	16.52	0.03	0.95	141.45	5.09	0.01	0.50	99.00	5.38	0.01	0.46	85.76

**Table A4:** Measurement and calculation of porosity parameters for woven fabrics using image analysis and reflectance and transmissive illumination: open area, average pore size, perimeter, and hydraulic diameter at different threshold algorithms and magnifications.

		Otsu								Yen							
		0.5				1				0.5				1			
Weave	Density	Open Area [%]	Size [mm^2^]	Perimeter [mm]	*D_h_* [μm]	Open Area [%]	Size [mm^2^]	Perimeter [mm]	*D_h_* [μm]	Open Area [%]	Size [mm^2^]	Perimeter [mm]	*D_h_* [μm]	Open Area [%]	Size [mm^2^]	Perimeter [mm]	*D_h_* [μm]
PW	20/15	21.39	0.09	1.19	287.44	21.30	0.07	1.15	246.23	24.81	0.09	1.18	305.27	25.12	0.08	1.13	267.19
	20/22	17.63	0.05	0.90	230.62	18.91	0.05	0.94	209.43	22.43	0.06	0.94	248.82	24.44	0.06	0.99	229.04
	29.3/15	16.05	0.05	0.85	219.99	16.79	0.04	0.85	191.09	20.21	0.05	0.91	229.08	21.21	0.04	0.88	196.59
	29.3/20	12.28	0.03	0.67	164.61	13.73	0.03	0.66	157.19	11.78	0.03	0.66	163.57	12.68	0.02	0.64	155.43
BW	20/15	22.70	0.12	1.20	385.29	20.84	0.07	0.86	331.53	26.12	0.11	1.20	360.20	26.21	0.08	1.01	311.69
	20/22	18.40	0.09	1.05	345.15	18.64	0.06	0.77	288.72	24.18	0.08	1.01	308.98	27.96	0.07	1.14	
	29.3/15	18.00	0.07	1.39	211.91	17.85	0.06	1.20	204.94	23.85	0.11	1.85	242.97	23.99	0.10	1.65	236.75
	29.3/20	14.36	0.05	0.93	212.77	14.90	0.04	0.86	200.48	23.08	0.09	1.53	234.80	23.67	0.07	1.29	219.61
R 4/2	20/15	24.75	0.15	1.59	367.21	24.80	0.13	1.50	344.22	29.26	0.14	1.45	373.22	30.40	0.10	1.20	345.75
	20/22	20.72	0.10	1.37	288.64	20.54	0.09	1.41	258.18	25.81	0.13	1.71	315.05	25.97	0.11	1.60	284.22
	29.3/15	15.13	0.06	1.02	224.18	15.22	0.05	0.92	210.27	22.48	0.06	1.01	224.72	23.30	0.05	0.91	209.94
	29.3/20	13.83	0.05	0.90	209.77	13.78	0.04	0.92	193.92	20.49	0.06	1.00	224.25	21.59	0.04	0.87	204.24
R 2/4	20/15	24.38	0.14	1.54	362.52	24.03	0.12	1.34	351.13	27.94	0.13	1.44	362.30	28.41	0.11	1.20	351.04
	20/22	20.03	0.11	1.29	329.89	19.87	0.09	1.17	291.24	25.50	0.10	1.24	331.30	27.21	0.08	1.09	285.52
	29.3/15	21.49	0.11	1.85	237.95	19.84	0.08	1.45	232.63	21.49	0.11	1.87	238.17	22.10	0.12	1.85	251.47
	29.3/20	16.79	0.06	1.11	225.11	16.97	0.05	1.00	196.98	20.96	0.10	1.58	241.99	20.94	0.07	1.35	211.98
T 1/3	20/15	24.44	0.08	1.17	268.77	26.11	0.08	1.14	264.95	24.86	0.08	1.18	270.74	28.03	0.08	1.22	274.16
	20/22	20.52	0.06	1.04	213.02	20.86	0.05	1.05	187.45	20.82	0.06	1.07	214.37	20.51	0.05	1.03	186.38
	29.3/15	25.66	0.08	1.40	230.74	24.01	0.06	1.28	195.04	18.54	0.05	0.98	185.10	15.01	0.03	0.76	143.91
	29.3/20	23.20	0.08	1.49	203.10	22.59	0.06	1.34	176.85	10.87	0.02	0.66	134.30	10.51	0.02	0.59	114.27
T 2/2	20/15	23.64	0.08	1.24	249.63	24.49	0.07	1.21	220.86	21.92	0.07	1.17	241.30	22.82	0.06	1.13	212.16
	20/22	22.25	0.06	1.10	218.95	22.32	0.06	1.09	205.17	20.02	0.05	0.98	208.40	20.26	0.05	0.98	195.40
	29.3/15	24.03	0.08	1.53	207.43	23.51	0.07	1.42	185.02	14.14	0.03	0.91	148.96	12.76	0.02	0.77	124.96
	29.3/20	21.43	0.06	1.41	177.26	21.14	0.05	1.32	159.06	10.80	0.02	0.68	123.57	10.40	0.02	0.62	106.47

## Appendix C

**Table A5:** Correlation coefficient between calculated parameters on real fabrics and parameters measured and calculated with image analysis.

Parameter Correlation	Weave	Transmission				Reflection				Transmission—Reflection			
		Otsu		Yen		Otsu		Yen		Otsu		Yen	
		0.5	1	0.5	1	0.5	1	0.5	1	0.5	1	0.5	1
Open area (fabrics)—Open area (image)	PW	0.988	0.997	0.984	0.994	0.988	0.988	0.985	0.987	0.975	0.983	0.890	0.865
	BW	0.956	0.951	0.984	0.946	0.991	0.991	0.990	0.983	0.938	0.933	0.966	0.693
	R 4/2	−0.994	−0.990	−0.999	−0.999	−0.995	−0.978	−0.996	−0.994	−0.989	−0.992	−0.995	−0.999
	R 2/4	−0.930	−0.984	−1.000	−0.999	−0.991	−0.972	−0.998	−0.988	−0.796	−0.910	−0.996	−0.981
	T 1/3	−0.465	−0.800	−0.962	−0.985	−0.529	−0.841	−0.953	−0.933	0.237	−0.334	−0.906	−0.982
	T 2/2	−0.909	−0.892	−0.999	−0.999	−0.741	−0.893	−0.995	−0.996	−0.390	−0.721	−0.961	−0.976
*D_h_* (fabrics)— *D_h_* (image)	PW	0.920	0.954	0.933	0.957	0.927	0.938	0.929	0.949	0.913	0.956	0.923	0.969
	BW	0.987	0.953	0.987	0.972	0.985	0.965	0.971	0.956	0.984	0.996	1.000	0.992
	R 4/2	0.998	0.983	0.988	0.971	0.985	0.992	0.984	0.984	0.998	0.989	0.996	0.999
	R 2/4	0.957	0.949	0.965	0.968	0.958	0.957	0.956	0.966	0.999	0.984	0.992	0.955
	T 1/3	0.788	0.903	0.921	0.948	0.918	0.922	0.898	0.906	0.628	0.726	0.912	0.928
	T 2/2	0.971	0.956	0.993	0.991	0.977	0.985	0.977	0.978	0.916	0.924	0.986	0.973
Equivalent pore diameter (fabrics)—*D_h_* (image)	PW	0.996	0.995	0.998	0.994	0.990	0.995	0.994	0.997	1.000	0.995	0.999	0.985
	BW	0.777	0.687	0.777	0.737	0.762	0.733	0.727	0.719	0.788	0.837	0.876	0.960
	R 4/2	0.871	0.845	0.822	0.817	0.821	0.927	0.826	0.912	0.922	0.941	0.858	0.885
	R 2/4	0.994	0.996	0.993	0.995	0.998	0.993	0.998	0.995	0.930	0.986	0.910	0.997
	T 1/3	0.919	0.957	0.987	0.990	0.968	0.990	0.999	0.993	0.898	0.917	0.996	0.987
	T 2/2	0.951	0.972	0.917	0.933	0.887	0.924	0.948	0.951	0.995	0.957	0.919	0.864
Porosity (fabrics)—*D_h_* (image)	PW	0.981	0.974	0.974	0.973	0.989	0.937	0.983	0.957	0.966	0.961	0.949	0.936
	BW	0.526	0.397	0.526	0.439	0.500	0.493	0.479	0.488	0.548	0.593	0.629	0.732
	R 4/2	0.792	0.768	0.735	0.737	0.736	0.868	0.744	0.854	0.854	0.880	0.774	0.808
	R 2/4	0.945	0.929	0.954	0.952	0.955	0.937	0.959	0.952	0.990	0.982	0.970	0.954
	T 1/3	0.864	0.900	0.949	0.957	0.916	0.956	0.978	0.963	0.906	0.889	0.988	0.949
	T 2/2	0.656	0.718	0.575	0.609	0.527	0.595	0.646	0.654	0.812	0.734	0.599	0.512

## References

[1] Zhu G., Kremenakova D., Wang Y., Militky J., Mishra R. (2015). Study on air permeability and thermal resistance of textiles under heat convection. Text. Res. J..

[2] Rajan T.P., Souza L.D., Ramakrishnan G., Zakriya G.M. (2016). Comfort properties of functional warp-knitted polyester spacer fabrics for shoe insole applications. J. Ind. Text..

[3] Otto J., Kaldenhoff E., Kirschner-Hermanns R., Mühl T., Klinge U. (2014). Elongation of textile pelvic floor implants under load is related to complete loss of effective porosity, thereby favoring incorporation in scar plates. J. Biomed. Mater. Res. A.

[4] Valencia R.A., García M.J., Bustamante J. (2018). A comparative computational study of blood flow pattern in exemplary textile vascular grafts. J. Text. Inst..

[5] Vatansever Bayramol D., Soin N., Dubey A., Upadhyay R.K., Priyadarshini R., Roy S.S., Shah T.H., Anand S.C. (2018). Evaluating the fabric performance and antibacterial properties of 3-D piezoelectric spacer fabric. J. Text. Inst..

[6] Azam F., Ahmad F., Ulker Z., Zafar M.S., Ahmad S., Rasheed A., Nawab Y., Erkey C. (2022). The Role and Applications of Aerogels in Textiles. Adv. Mater. Sci. Eng..

[7] Kostajnšek K., Zupin Ž., Hladnik A., Dimitrovski K. (2021). Optical Assessment of Porosity Parameters in Transparent Woven Fabrics. Polymers.

[8] Swery E.E., Allen T., Kelly P. (2016). Automated tool to determine geometric measurements of woven textiles using digital image analysis techniques. Text. Res. J..

[9] Tàpias M., Ralló M., Escofet J. (2011). Automatic measurements of partial cover factors and yarn diameters in fabrics using image processing. Text. Res. J..

[10] Meškuotienė A., Dargienė J., Domskienė J. (2015). Metrological performance of the digital image analysis method applied for investigation of textile deformation. Text. Res. J..

[11] Ragab A., Fouda A., El-Deeb H., Abou-Taleb H. (2017). Determination of Pore Size, Porosity and Pore Size Distribution of Woven Structures by Image Analysis Techniques. J. Text. Sci. Eng..

[12] Giacalone V., Civilini V., Audenino A.L., Terzini M. (2023). Quantifying mesh textile and effective porosities: A straightforward image analysis procedure for morphological analysis of surgical meshes. Comput. Methods Programs Biomed..

[13] Kočevar T.N., Gabrijelčič Tomc H. (2018). Analysis of methods used for texture preparation for 3D visualisation of fabric porosity. J. Text. Inst..

[14] (1996). Textiles—Determination of Thickness of Textiles and Textile Products.

[15] (1996). Textiles—Woven Fabrics—Determination of Mass per Unit Length and Mass per Unit Area.

[16] (1999). Textiles—Woven fabrics—Construction—Methods of analysis—Part 2: Determination of Number of Threads per unit Length (ISO 7211–2:1984 modified).

[17] Zupin Ž., Hladnik A., Dimitrovski K. (2012). Prediction of one-layer woven fabrics air permeability using porosity parameters. Text. Res. J..

[18] Jakšić D., Jakšić N. (2007). Assessment of Porosity of Flat Textile Fabrics. Text. Res. J..

[19] Dubrovski P.D. (2000). Volume Porosity of Woven Fabrics. Text. Res. J..

[20] Scheidegger A.E. (1974). The Physics of Flow through Porous Media.

[21] Rout P.K., Singh M.K. (2023). Porosity determination of textile fabrics: A novel mathematical approach and experimental validation. Mater. Today Commun..

[22] Dimitrovski K., Novak I. (2019). Equivalent average diameter of pores—Definition and determination. Textile Science and Economy 2019 French—Croatian Forum.

[23] NumPy. https://numpy.org/.

[24] OpenCV. https://opencv.org/.

[25] Otsu N. (1979). A Threshold Selection Method from Gray-Level Histograms. IEEE Trans. Syst. Man. Cybern..

[26] Yen J.C., Chang F.J., Chang S. (1995). A new criterion for automatic multilevel thresholding. IEEE Trans. Image Process..

[27] Begum M.S., Milašius R. (2022). Factors of Weave Estimation and the Effect of Weave Structure on Fabric Properties: A Review. Fibers.

